# Comparative Analysis of Rotary Systems in Curved Root Canals: Evaluation of Wear, Transportation, and Centering Capacity

**DOI:** 10.3390/dj14020092

**Published:** 2026-02-05

**Authors:** Siri Paulo, Pedro Zagalo, Beatriz Louro, Ricardo Jorge Teixo, José Pedro Martinho, Tiago Nóbrega, Anabela Paula, Carlos Miguel Marto, Diogo Fonseca, Manuel Marques Ferreira

**Affiliations:** 1 Institute of Endodontics, Faculty of Medicine, University of Coimbra, 3000-548 Coimbra, Portugal; pedrozagalo50@gmail.com (P.Z.); beatriz_louro@hotmail.com (B.L.); josepedromartinho@gmail.com (J.P.M.); tiagonbrg@gmail.com (T.N.); diogo.fonseca@ff.uc.pt (D.F.); mmferreira@fmed.uc.pt (M.M.F.); 2 Area of Environment, Genetics and Oncobiology (CIMAGO), Faculty of Medicine, Coimbra Institute for Clinical and Biomedical Research (iCBR), University of Coimbra, 3000-548 Coimbra, Portugal; ricardo.teixo@fmed.uc.pt (R.J.T.); appaula@fmed.uc.pt (A.P.); 3 Center for Innovative Biomedicine and Biotechnology (CIBB), University of Coimbra, 3000-548 Coimbra, Portugal; 4 Clinical Academic Center of Coimbra (CACC), University of Coimbra, 3000-354 Coimbra, Portugal; 5 Institute of Integrated Clinical Practice, Faculty of Medicine, University of Coimbra, 3000-548 Coimbra, Portugal; 6 Centre for Mechanical Engineering, Materials and Processes (CEMMPRE), University of Coimbra, 3000-548 Coimbra, Portugal; 7 Laboratory for Evidence-Based Sciences and Precision Dentistry, University of Coimbra, 3000-548 Coimbra, Portugal; 8 Institute of Biophysics, Faculty of Medicine, University of Coimbra, 3000-548 Coimbra, Portugal; 9 Institute of Experimental Pathology, Faculty of Medicine, University of Coimbra, 3000-548 Coimbra, Portugal; 10 Faculty of Pharmacy, University of Coimbra, 3000-548 Coimbra, Portugal

**Keywords:** curved root canals, wear, transportation, centering ability, ProTaper Next, TruNatomy, ProTaper Ultimate, RACE EVO

## Abstract

**Background/Objectives:** Root canal instrumentation has a crucial role in the success of endodontic treatment. However, management of curved root canals remains a challenge. This study aimed to compare the performance of four rotatory file systems, ProTaper Next, TruNatomy, ProTaper Ultimate and Race Evo, in terms of wear, transportation and centering capacity, in curved root canals. **Methods:** A total of 150 human tooth roots were selected, divided based on the degree of curvature, and then distributed into four experimental groups according to the rotary system used. Cone beam computed tomography images were obtained before and after instrumentation, and values were measured with ImageJ software. **Results:** Regarding root canal wear, the TruNatomy system displayed the lowest wear values, and the Race Evo system showed a tendency for greater wear. For transportation, TruNatomy and Race Evo had the lowest transportation, indicating a higher respect for the root canal’s original anatomy. For centering ability, Race Evo and ProTaper Ultimate displayed values closer to perfect centering compared to other systems. **Conclusions:** Overall, TruNatomy was confirmed as a more conservative system, Race Evo with a tendency for greater wear even though with a higher respect for root canal original anatomy. Race Evo and ProTaper Ultimate showed better centering ability.

## 1. Introduction

Endodontic treatment aims to remove necrotic, vital, or infected pulp tissue in order to receive the filling material, to keep the tooth in the arch, and restore its function [1].

The root canal preparation phase in endodontic treatment aims to achieve adequate disinfection and preparation of the canal system, for subsequent filling with an inert material [2]. According to the literature, correct instrumentation must respect the original base morphology of the root canal, without weakening the tooth or displacing the apical foramen from its original anatomical position, giving a progressive conicity, from the apical to the cervical region [3,4,5] and preserving its original anatomy, position, and centralization [3,6,7,8,9,10].

The complexity of root canal anatomy makes each tooth unique [3]. To prevent procedural errors, which can lead to unsuccessful endodontic treatment, the anatomy and complexity of the root canal system, as well as the degree of curvatures, must be assessed pre-operatively [11].

In curved canals, disinfection and root canal instrumentation constitute an even greater challenge, as curvatures of different degrees can lead to several undesirable accidents [11,12]. In this type of root canal, there is increased difficulty in keeping the long axis centered in the canal, and there is an increased risk of excessive wear of the canal walls, deviations, perforations, apical transport, step formation, and file fracture [6,12,13,14,15,16,17,18]. Thus, factors such as instrumentation technique, flexibility, and diameter of endodontic instruments, location of the apical foramen, and dentin hardness can influence the result of instrumentation of curved canals [14].

In fact, root canals have primary and secondary curvatures that require the instruments to have a flexibility that reduces the internal forces inherent to the instrument itself, which tend to restore it to its original straight position. These forces cause the file to move towards outside the central axis of the root canal, causing canal transportation, thus leading to excessive wear in some areas and under instrumentation in others [15].

Stainless steel hand files are the most used instruments in endodontics. However, these have disadvantages such as their reduced flexibility, higher transport rate, and lower rate of centralization of the root canal, thus being associated with a greater probability of undesirable intraoperative accidents [19,20]. The disadvantages associated with these instruments have led to the search for new materials and manufacturing methods [21].

In this context, rotary instruments made of nickel/titanium (NiTi) alloys were introduced onto the market, showing greater flexibility and greater resistance to torsional and cyclic fatigue [15]. Continuous rotation NiTi systems have also shown a greater capacity to preserve the original canal anatomy, especially in curved canals, presenting better performance regarding canal centralization and transportation. Consequently, they also reduce the possibility of iatrogenic events occurring during the instrumentation of curved canals, allowing for safer, more focused preparation and with a better prognosis than with the use of manual stainless-steel instruments [6,7,8,9,10,15,17,18,19,20,22].

Within the several generations of NiTi alloy instruments, the ProTaper Next continuous rotation system (PTN) has been one of the most used over the years, despite not being one of the most recent. Among the currently available rotary NiTi instruments, the TruNatomy system (TN), the ProTaper Ultimate system (PTU), and the RACE EVO system (RE) are among the systems most aimed at approaching canals with curvatures.

The literature on these rotary systems is still scarce, particularly regarding their suitability for curved root canals. Therefore, the aim of this work was to evaluate the effects of root canal instrumentation with the various rotary systems that were mentioned (PTN, TN, PTU, and RE) on curved root canals. For that purpose, we compared wear, canal transportation, and centering ability based on cone beam computed tomography (CBCT) imaging.

The null hypothesis was that there was no statistically significant difference between the various systems in relation to root canal wear, transportation, and centering ability after rotary instrumentation, both in straight root canals or in root canals with moderate and severe curvatures.

## 2. Materials and Methods

### 2.1. Teeth Selection

A total of 97 mono and multiradicular teeth (corresponding to 150 roots), extracted due to reasons not related to this study, were selected. The following were considered as exclusion criteria: teeth with root fissures or fractures, pulp chamber calcifications, internal root resorptions, previous endodontic treatment, and teeth with open apices.

The study was conducted in accordance with the Declaration of Helsinki and approved by the Ethics Committee of the Faculty of Medicine of the University of Coimbra and was approved under the number CE-086/2019.

After removal of periodontal tissue remains and debris attached to the root surface, teeth were placed in 5% sodium hypochlorite for 1 h and then preserved in saline solution. Teeth were then numbered and included in putty silicone (Aquasil Soft Putty, Dentsply Sirona, Konstanz, Germany), in order to be radiographed with the retroalveolar technique in the buccal-lingual direction.

The division of teeth roots was performed according to the Schneider method [23], where the degree of curvature was determined by the angle formed between two lines, the first being drawn parallel to the canal long axis and the second being designed from the apical foramen to the first line, at the point where it no longer follows the long axis of the tooth.

**Figure 1:**
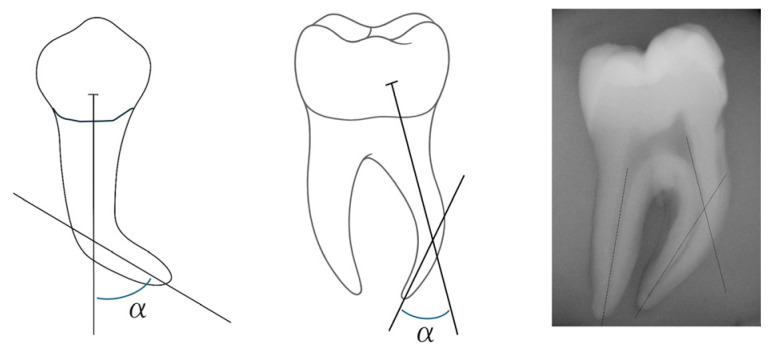
Illustration of the Schneider method of evaluation of root curvature (α angle formed between the line parallel to the canal long axis and the line from the apical foramen to the first line).

The formed angle was calculated using the ImageJ program, as shown in Figure 1. Then, roots were divided into “straight” (curvatures less than 10°, *n*= 36), “moderate curvature” (curvatures from 10° to 25°, *n* = 54) and “severe curvature” (curvatures greater than 25°, *n* = 60), allowing the evaluation of results to be carried out depending on the degree of curvature [23]. Roots were randomly distributed into groups with a similar average curvature. Markings were made on teeth roots at 3 and 5 mm from the apex with heated gutta-percha, in order to obtain references to the locations where measurements, before and after instrumentation, would be carried out.

### 2.2. Chemo-Mechanical Preparation of Canals

Preparation of the endodontic access cavity began with a spherical diamond turbine drill, and the permeability of the canal was confirmed; compensatory drilling was then carried out. The length of the root canal was determined by the visibility of the tip of a K10 file (Dentsply Maillefer, Ballaigues, Switzerland) at the apex, and the file rubber stop was adjusted. The length of the root canal was measured with the aid of an endodontic ruler, and the working length (WL) was determined by subtracting 0.5 mm from the length of the canal.

Before starting mechanical instrumentation, root canals were permeabilized again with a K10 file, as with all instruments. During instrumentation, canals were irrigated with 2.5% sodium hypochlorite (NaOCl) between each instrument and permeabilized with a K10 file (WL + 0.5 mm), as the files were cleaned with damp gauze. EDTA-based gel (Glyde, Dentsply Maillefer, Ballaigues, Switzerland) was used as a canal lubricant.

In the PTN system group (Dentsply Maillefer, Ballaigues, Switzerland), canals were instrumented with the ProGlider file (016/0.02), with the X1 (17/variable taper) and X2 (25/variable taper) files to the WL (Table 1).In the TN system group (Dentsply Maillefer, Ballaigues, Switzerland), the glidepath was reinforced with the TN Glider file (17/0.02) to the WL, and the canal was instrumented with the TN Prime shaping file (26/0.04) to the WL (Table 1).

In the RE system group (FKG Dentaire SA, La Chaux de Fonds, Switzerland), RE1 files (15/0.04) were used to reinforce the glidepath, and RE2 (25/0.04) was advanced to the WL (Table 1).In the PTU system group (Dentsply Maillefer, Ballaigues, Switzerland), canals were instrumented mechanically with the Slider (16/0.02), Shaper (20/0.04), and Finisher F1 (20/0.07) to the WL (Table 1).

The files were selected following the protocol of their respective rotary file systems, but also to achieve some homogeneity in taper values, so that they would be eligible for comparison of wear, transportation, and centering ability (Table 2).

Mechanical instrumentation was performed with an electric motor (WaveOne^TM^; Dentsply Maillefer, Ballaigues, Switzerland), in continuous rotation:

- At 250 rpm, with a torque of 2 Ncm, in group 1 (PTN);
- At 500 rpm, with a torque of 1.6 Ncm, in group 2 (TN);
- At 800 rpm, in continuous rotation, and with a torque of 1.5 Ncm in group 4 (RE);
- At 400 rpm, in continuous rotation, and with a torque of 4 Ncm in group 3 (PTU).

At the end of the instrumentation, apical permeabilization was checked, and canals were irrigated with 2 mL of 2.5% NaOCl.

### 2.3. Evaluation

To compare the efficiency of rotary instrumentation systems, several methods have been used, such as radiographic superimposition, cone beam computed tomography (CBCT), and microcomputed tomography [24,25]. In this study, we used CBCT images of the platforms that were obtained with the i-CAT Vision™ scanner (Imaging Sciences International, Hatfield, PA, USA) in the same position and with the same exposure parameters before and after instrumentation. These parameters include a field of view of 8 × 8 cm, a resolution of 0.125 Voxel, with an acquisition time of 26.9 s, and an axial slice with a thickness of 0.13 mm.

### 2.4. Image Analysis

**Figure 2:**
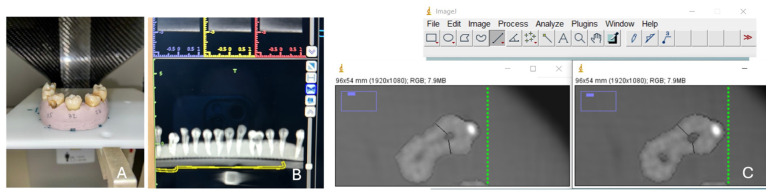
(**A**) Positioning of teeth for CBCT; (**B**) all teeth in the CBCT image; (**C**) ImageJ software distance measurements before and after instrumentation, according to the method of Gambill et al. [21] (black lines represent the distance from the wall of the canal to the external wall of the root in the mesial and distal directions).

To evaluate differences in canal dimension before and after instrumentation, we used the method described by Gambill et al. [21]. Thus, the shortest distances were measured in the CBCT images, from the wall of the non-instrumented canal to the external wall of the root in the mesial (M_1_) and distal (D_1_) directions, comparing them with the same measurements in the images with the instrumented canals, as M_2_ corresponds to the measurement in the mesial part and D_2_ in the distal part after instrumentation. Measurements were carried out using the ImageJ program (ImageJ v1.53 series, NIH, Bethesda, MD, USA), as shown in Figure 2.

The values were measured in the middle third of the canal (5 mm from the apex) and in the apical third (3 mm from the apex), with the ImageJ program, as shown in Figure 2. Measurements were carried out and confirmed by four independent evaluators (P.Z., B.L., D.F., and S.P.).

The wear, transport, and centralization of the canal were calculated, after measurements, according to the following formulas:

Total mesio-distal wear of the canal = (M_1_ − M_2_) + (D_1_ − D_2_) (the higher the value, the greater the total mesio-distal wear of the root canal)

Canal transportation = (M_1_ − M_2_) − (D_1_ − D_2_) (a value of 0 indicates no transport, a positive value indicates canal transport towards the external curvature of the root, and a negative value indicates canal transport towards the internal curvature of the root)

Canal centering = (M_1_ − M_2_)/(D_1_ − D_2_), if (D_1_ − D_2_) > (M_1_ − M_2_) or = (D_1_ − D_2_)/(M_1_ − M_2_), if (M_1_ − M_2_) > (D_1_ − D_2_) (perfect centering occurs when the result is 1, and the centering ability of the instrument decreases as the ratio approaches 0)

The obtained measurements and the calculations of the formulas representing these parameters were recorded in Microsoft Excel tables (Microsoft Corp, Redmond, WA, USA) and verified.

### 2.5. Statistical Analysis

Statistical analysis was performed using IBM^®^ SPSS^®^ 28.0 software (IBM Corporation, Armonk, NY, USA), and graphical representation was performed using GraphPad Prism 9.0 software (Dotmatics, Boston, MA, USA). The results for canal wear were generally presented as median (minimum; maximum). Data normality was analyzed using the Shapiro-Wilk test, while homogeneity of variances was assessed using Levene’s test. Parametric tests (one-way analysis of variance or ANOVA) were used when a normal distribution of quantitative variables was observed, and non-parametric tests (Kruskal–Wallis with Dunn’s multiple comparisons) were used when normality was not verified. A significance level of 0.05 was considered.

## 3. Results

Results are presented according to the parameters that were evaluated, i.e., root canal wear, transportation, and centering ability, and were primarily organized by the type of canal: straight canals, canals with moderate curvature, and canals with severe curvature.

### 3.1. Canal Wear

**Figure 3:**
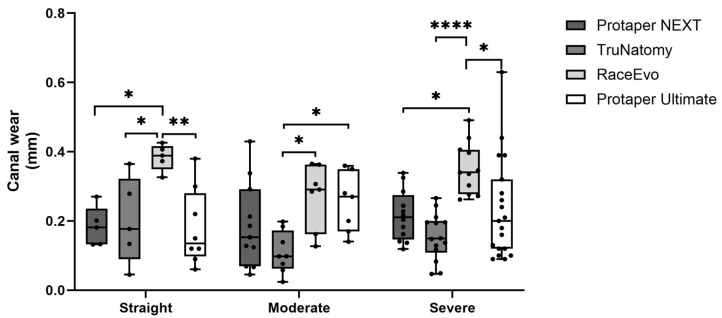
Total mesio-distal wear at 3 mm in straight canals, as well as canals with moderate and severe curvatures. Box plot displays median and interquartile ranges. Statistical significance is indicated by asterisks (* *p* < 0.05, ** *p* < 0.01, and **** *p* < 0.0001).

**Figure 4:**
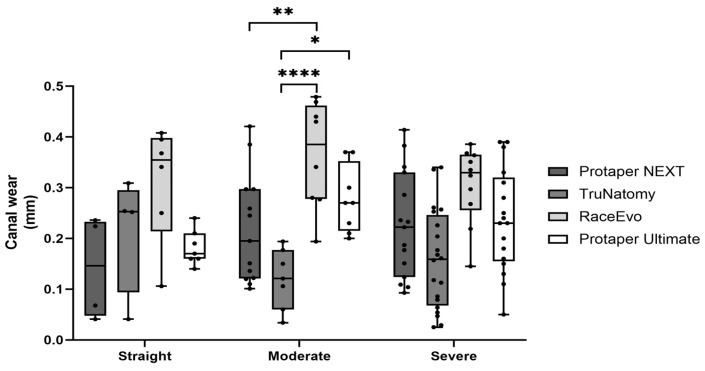
Total mesio-distal wear at 5 mm in straight canals, as well as canals with moderate and severe curvatures. Box plot displays median and interquartile ranges. Statistical significance is indicated by asterisks (* *p* < 0.05, ** *p* < 0.01, and **** *p* < 0.0001).

Root canal wear was assessed as total mesio-distal wear, where the higher the value is, the greater the wear. Results for measurements at 3 mm from the apex are presented in Figure 3 and Table 3, and for 5 mm in Figure 4 and Table 4.

#### 3.1.1. Straight Canals

At 3 mm from the apex, RE showed a significantly higher wear [0.389 mm (0.326; 0.426)], almost double when compared to PTN (*p* = 0.015 vs. RE), TN (*p* = 0.028 vs. RE), and especially PTU, which was the system with the lowest values [0.135 mm (0.060; 0.380), *p* = 0.006 vs. RE]. No statistically significant differences were observed between PTU, PTN, and TN systems.

At 5 mm, RE showed a tendency for higher wear [0.335 mm (0.106; 0.408)] when compared to the other systems, but no statistically significant differences were observed.

Overall, the RE system exhibited the highest wear in straight canals at 3 mm from the apex, as other systems showed similar results at this reference point.

#### 3.1.2. Canals with Moderate Curvatures

At 3 mm from the apex, RE [0.291 mm (0.127; 0.365)] exhibited the highest wear values, followed by PTU [0.270 mm (0.140; 0.360)] system. PTN [0.153 mm (0.045; 0.430)] and TN [0.098 mm (0.024; 0.198), *p* = 0.015 vs. RE; *p* = 0.037 vs. PTU] were the systems with the lowest wear values.

At 5 mm, RE also showed a higher wear [0.386 mm (0.194; 0.479)], standing out from the other systems, followed by PTU [0.270 mm (0.200; 0.370)], PTN [0.195 mm (0.101; 0.421), *p* = 0.007 vs. RE] and TN [0.121 mm (0.034; 0.194), *p* < 0.0001 vs. RE and *p* = 0.012 vs. PTU].

Overall, the RE system exhibited the highest wear in moderate curvatures, and the TN system exhibited the lowest.

#### 3.1.3. Canals with Severe Curvatures

At 3 mm from the apex, RE showed higher wear values [0.340 mm (0.262; 0.491)], with statistically significant differences when compared to PTN [0.211 mm (0.119; 0.339), *p* = 0.044], PTU [0.190 mm (0.090; 0.630), *p* = 0.011] and TN [0.149 mm (0.047; 0.266), *p* < 0.0001].

At 5 mm, RE maintained a tendency to exhibit a higher wear [0.330 mm (0.145; 0.386)] compared to other systems [PTU 0.236 mm (0.050; 0.390); PTN 0.222 mm (0.414; 0.093); TN 0.159 mm (0.025; 0.340)], but with no statistically significant differences.

Overall, RE showed the highest wear values at 3 mm from the apex, as other systems showed similar results at this reference point.

### 3.2. Canal Transportation

**Figure 5:**
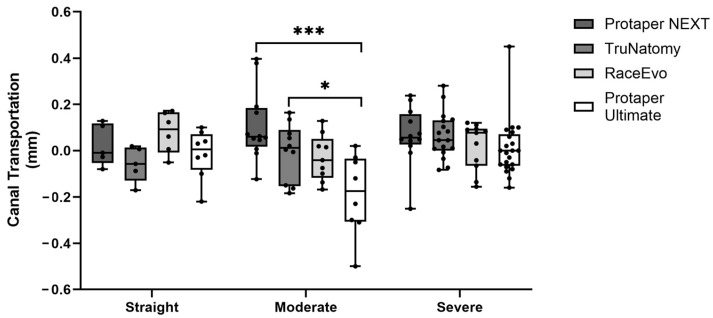
Canal transportation at 3 mm in straight canals, as well as canals with moderate and severe curvatures. Box plot displays median and interquartile ranges. Statistical significance is indicated by asterisks (* *p* < 0.05 and *** *p* < 0.001).

**Figure 6:**
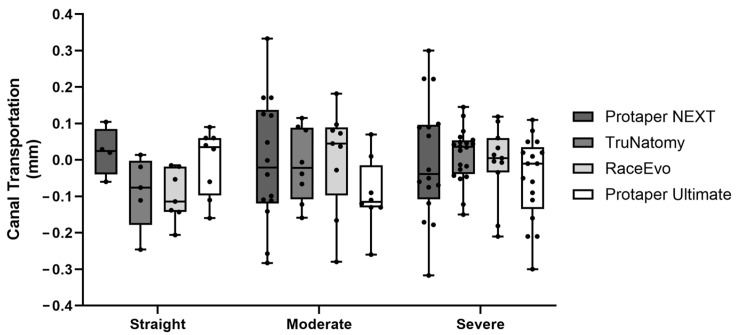
Canal transportation at 5 mm in straight canals, as well as canals with moderate and severe curvatures. Box plot displays median and interquartile ranges.

Root canal transportation was also assessed, as a value of 0 indicates no transportation, a positive value indicates transportation towards the external curvature, and a negative value indicates transportation towards the internal curvature. Results for 3 mm are presented in Figure 5 and Table 5, and for 5 mm in Figure 6 and Table 6.

#### 3.2.1. Straight Canals

At 3 mm, TN (−0.057 mm (−0.171; 0.019)), as well as PTN [−0.009 mm (−0.080; 0.128)], showed a tendency for canal transportation towards the internal curvature, although with no statistically significant difference (*p* = 0.164). RE [0.092 mm (−0.051; 0.172)] and PTU [0.005 mm (−0.220; 0.100)] showed a tendency for transportation towards the external curvature, also with no significant differences. No significant differences were observed at 5 mm.

#### 3.2.2. Canals with Moderate Curvatures

At 3 mm from the apex, canal transportation had opposite results compared with straight canals. PTN [0.060 mm (−0.123; 0.369)] and TN [0.012 mm (−0.184; 0.164)] showed a tendency for canal transportation to the outer side of the curvature, with a statistically significant difference when compared with PTU [−0.175 mm (−0.500; 0.02)], which had a tendency to transport canal to the inner part of the curvature (*p* = 0.0003 vs. PTN, *p* = 0.042 vs. TN). At 5 mm, statistically significant differences were not observed.

#### 3.2.3. Canals with Severe Curvatures

At 3 mm, PTN, TN, and RE showed a slight tendency for canal transportation to the outer side of the curvature and PTU to the inner part, but without statistically significant differences. No significant differences were observed at 5 mm.

### 3.3. Canal Centering Ability

**Figure 7:**
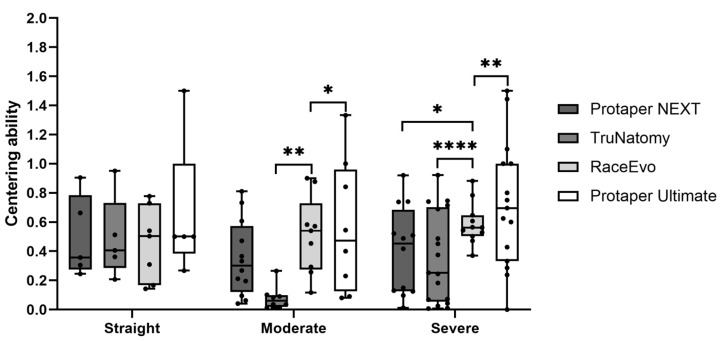
Centering ability at 3 mm in straight canals, as well as canals with moderate and severe curvatures. Box plot displays median and interquartile ranges. Statistical significance is indicated by asterisks (* *p* < 0.05, ** *p* < 0.01, and **** *p* < 0.0001).

**Figure 8:**
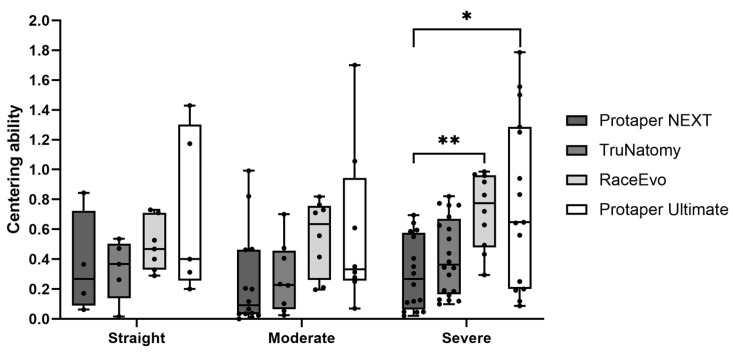
Centering ability at 5 mm in straight canals, as well as canals with moderate and severe curvatures. Box plot displays median and interquartile ranges. Statistical significance is indicated by asterisks (* *p* < 0.05 and ** *p* < 0.01).

Root canal centering ability was also evaluated, as perfect centering occurs with a result of 1, whereas it decreases when the ratio approaches 0 (zero). Results for 3 mm are presented in Figure 7 and Table 7, and for 5 mm, in Figure 8 and Table 8.

#### 3.3.1. Straight Canals

At 3 mm from the apex, RE [0.504 mm (0.143; 0.777)] and PTU [0.500 mm (0.260; 1.500)] showed an intermediate tendency towards centering, when compared to a lower tendency from PTN [0.357 mm (0.245; 0.905)] and TN [0.406 mm (0.207; 0.951)], although without statistically significant differences.

At 5 mm, RE had the highest value of centering ability with 0.468 mm (0.329; 0.731), followed by PTU [0.400 mm (0.313; 1.429)]. The lowest values were from TN [0.367 mm (0.016; 0.537)] and PTN [0.269 mm (0.063; 0.844)]. No statistically significant differences were observed.

#### 3.3.2. Canals with Moderate Curvatures

At 3 mm, TN [0.080 mm (0.013; 0.265)] had the lowest tendency towards centering, followed by PTN [0.301 mm (0.041; 0.811)]. With an intermediate tendency, PTU [0.473 mm (0.080; 1.330)] was then followed by RE [0.541 mm (0.117; 0.900), *p* = 0.004 vs. TN and *p* = 0.015 vs. PTU]. Nevertheless, all are far from the value of 1 (the ideal value of centering ability).

At 5 mm, PTN had the lowest value [0.091 mm (0.000; 0.992)], followed by TN with 0.229 mm (0.025; 0.700), PTU with 0.331 mm (0.071; 1.700), and then RE with 0.634 mm (0.196; 0.818). No statistically significant differences were observed.

#### 3.3.3. Canals with Severe Curvatures

At 3 mm, PTU [0,720 mm (0.000; 1.500), *p* = 0.01 vs. RE] showed the greater centering ability, followed by RE with 0.563 mm (0.371; 0.881) and by PTN [0.452 mm (0.012; 0.920), *p* = 0.021 vs. RE]. TN stands out with the lowest centering ability [0.252 mm (0.007; 0.921), *p* = 0.0001 vs. RE].

At 5 mm, RE [0,774 (0.295; 0.986)] had the best centering ability, with PTU following it [0.647 mm (0.087; 1.786)]. TN [0.364 mm (0.099; 0.821)] and PTN [0.268 mm (0.022; 0.695), *p* = 0.008 vs. RE and *p* = 0.019 vs. PTU] maintained the lowest values.

## 4. Discussion

Several generations of files have emerged over the years, with the manufacturers improving the characteristics and type of alloys to facilitate a more effective approach to curved canals, preventing risks that arise during instrumentation [26].

To assess the performance of the files, all types of natural teeth were used, from single-rooted to multi-rooted, because they allow us to study the performance of endodontic instruments on different groups of teeth and on natural dentin, recreating the most realistic clinical conditions when compared with standardized artificial canals [27]. For a better simulation of clinical practice, the dental crowns were maintained [28].

CBCT, the radiographic method after instrumentation, allows a three-dimensional (3D) assessment of canal changes after instrumentation, in a precise and reproducible manner, specifically the reduction in intra-canal dentin thickness [6,9,21,28,29]. Furthermore, it enables a more accurate analysis of the canal anatomy without overlapping and image distortion [11,28]. Measurements were taken in the apical and middle thirds of the root canals, at 3 and 5 mm, respectively, as these regions are known to present accentuated curvatures, which increase the likelihood of iatrogenic events [18,25].

Regarding the calculations described by Gambill et al. [21], chosen to calculate the wear, transportation, and centering ability, these are the most referred to in the literature and allow these parameters to be evaluated effectively, through images obtained by CBCT [6,7,9,10,16,17,18,19,20,21,30,31].

In our study, we compared four continuous rotation systems: PTN, TN, PTU, and RE. The PTN is still one of the most used systems and, as a standard system, it is used as a reference in this type of research [2,4,13,32]; therefore, it was selected as a control in this study.TN, PTU, and RE instruments are increasingly recognized as some of the most suitable for approaching curved canals [33,34,35,36,37]; PTN files, manufactured in M-Wire, were developed with an eccentric rectangular cross-section to reduce the points of contact with the canal walls, thus reducing instrument fatigue [15,32]. The TN files have super-elastic properties and an eccentric parallelogram cross-section. The lower taper, greater flexibility, and less memory of these files aim at a higher preservation of tooth structure and of canal geometry, particularly relevant in curved canals [33,34].

RE files, with two distinct file sequences (4% and 6% taper), are manufactured from NiTi alloys with heat treatment and electro-polishing of the surface and have a triangular cross-section with alternating cutting edges, providing high flexibility, resistance to cyclic fatigue, and reduction of the screw-in effect [35,36].

The PTU is composed of eight instruments with an eccentric parallelogram shape manufactured with three different heat treatments—M-wire (Slider), Gold-wire (SX, Shaper, F1, F2, F3), and Blue heat-treated wire (FX and FXL)—with the goal of increasing flexibility and resistance to cyclic fatigue [37]. Of note, the three finisher instruments (F1, F2, and F3) have a fixed taper up to D3 and a decreasing taper from D4 to D16, in contrast with the increasing taper design of the PTU Shaper file. This design significantly improves flexibility and reduces potential locking in the canal that the increasing taper would favor.

There is still a scarcity of literature about their performance, especially on TN, RE, and PTU files, in relation to wear, transportation, and centering ability, particularly in curved canals. In our study, we compared the performance of these systems in straight canals or canals with a slight curvature (<10°), canals with moderate curvatures (10–25°), and canals with severe curvatures (>25°). As the files from the several systems present taper differences, files with approximate diameters in D3 and D5 were chosen as the last files in the instrumentation sequence. These files, in D0, D3, and D5, have diameters of 0.25–0.43–≅0.56 (PTN X2), 0.26–0.38–0.46 (TN Prime), 0.25–0.37–0.45 mm (RE R2), 0.20–0.41–0.55 mm (PTU F1), being the most compatible combination between the systems.

The transversal diameter, at 5 mm, of the last file used (TN prime) is 0.46 mm, lower than PTN X2 with ≅0.56 mm and PTU F1 with 0.55 mm. At 3 mm, the differences were lower, with 0.05 mm of difference in transversal diameter instead of 0.1 mm, as occurred at 5 mm. The transversal diameter of the last file used (RE R2), at 5 mm and 3 mm, was 0.46 mm and 0.38 mm, respectively, similar to TruNatomy, and lower than the referred PTN X2 and PTU F1.

Total mesio-distal wear represents an important measurement of dentin removal, with a potential impact on the predisposition to root fracture [38]. Overall, the RE group exhibited the highest wear in all canal configurations, particularly in the apical third (3 mm), as the TN group displayed the lowest wear in canals with moderate or severe curvatures. Given the fact that RE R2 files have a lower transversal diameter, at 3 mm and 5 mm, similar to TN prime, and since RE R2 promotes the higher wear and TN prime the lower wear, these findings may be attributed to the specific kinematic characteristics of the RE files. Regarding the transversal diameter, as was expected, the PTU was the second with the highest wear at 3 mm and 5 mm of moderate curvatures, and in severe curvatures, the second with the highest wear was PTN at 3 mm and PTU at 5 mm. In terms of canal transportation, the PTU group showed a tendency for transportation to the inner side of the curvature, whereas the PTN and TN groups showed the opposite, at 3 mm. Importantly, canal transportation in the apical third of curved canals equal to or greater than 0.3 mm may lead to a compromise of the apical sealing [39]. In this study, none of the groups showed a transportation greater than or equal to 0.3 mm, meaning that all file systems respect the canal’s original anatomy and thus can be considered safe to use in clinical practice.

Regarding centering ability, the RE and PTU groups showed the highest centering ability (RE with significantly higher values in moderate curvatures at 3 mm and PTU with significantly higher values in severe curvatures at 3 mm). Interestingly, the TN group showed significantly lower centering values in moderate and severe curvatures at 3 mm. This can be explained by the existence of extremely low values of wear on one side of the canal, leading to very low values that may not necessarily correspond to a decentralization of the canal, but rather to the fact that wear is very conservative on one side of the canal.

Overall, the RE group showed higher values of wear but with higher centering ability in moderate curvatures. The PTU group showed lower values of wear compared with RE and higher centering ability in severe curvatures. The TN group displayed the lowest values of wear in moderate and severe curvatures, but lower centering values in these curvatures. The PTN group displayed intermediate wear values but low centering ability, similar to TN.

For PTN files, several studies have shown a good centering ability in curved canals, when compared with WaveOne [40,41], ProTaper Universal [41], and TRU Shape [25]. However, a lower centering ability has been shown in comparison with instruments with higher flexibility, e.g., HyFlex CM and EDM systems [15], which is in accordance with our results. Potential discrepancies in results could also be explained by the different methodologies used, such as the evaluation method, the type of tooth, whether it is a natural tooth or an acrylic block, the comparison with reciprocating systems, and the instrumentation technique [28].

As for RE files in curved canals, other authors have reported lower canal transportation, lower wear, or higher centralization values compared to ProTaper Gold [30,42]. Also, a lower tendency to centralization at 3 mm and higher at 5 mm has been reported in comparison with EdgeSequel in curved canals [43]. Here, we observed a high centering ability, particularly in moderate curvatures, which is especially in line with the study by Mustafa [42]. The differences in results regarding wear may be due to different file systems that were compared, e.g., we compared RE with PTU, TN, and PTN, whereas Islam et al. [30] compared RE with ProTaper Gold (the predecessor of PTU).

Regarding TN, significantly lower canal transportation in curved canals has been reported compared to Reciproc Blue [44] and ProTaper Gold [45]. Compared to PTN, similar canal transportation has been shown [46,47]. However, results on centering ability are not consistent, as the study by Siraparapu et al. [46] showed higher centering ability in the apical part with TN compared to PTN, while other studies showed similar centering ability in curved canals [47,48]. In our study, we observed lower centering values for TN in the apical third of moderate and severe curvatures.

Concerning PTU files, Alovisi et al. [49] recently showed a similar performance in terms of centering ability compared to ProTaper Gold and PTN in mesial canals of mandibular molars, using micro-CT and Finite Element Analysis. In our study, we focused on curved canals, and the PTU group showed lower values of wear compared with RE and higher centering ability in severe curvatures.

In a micro-CT study by Gandhi et al. [50], the PTU system showed superior preservation of tooth structure with less canal enlargement while maintaining effective instrumentation, when compared to PTN. Compared to TN, both PTU and TN provided high preservation of the pericervical dentin [51]. However, the study by Lopes et al. [52] suggested PTU could lead to a less conservative preparation in mesial canals of mandibular molars, which was not verified in our study, which also considered PTU conservative, despite the higher taper.

Some limitations to our study should be acknowledged, namely the following: (a) although CBCT is a useful tool for canal anatomy assessment, it may give less precise measurements when compared to a method like micro-CT which has also been used in some studies; (b) only four mechanical file systems were used, as many others are available on the market, and further studies should include other systems. Regarding the PTU, our final instrument was the F1 file rather than the F2 file, which is more common clinically. Although this could represent a limitation in our study, some authors have suggested the use of smaller martensitic files in more challenging anatomies [53], and also, we aimed at obtaining the most compatible combination of diameters between the several systems.

## 5. Conclusions

Overall, TruNatomy was confirmed as a more conservative system in curved canals, even though Race Evo, with a tendency for greater wear, has a higher respect for the root canal’s original anatomy. Race Evo and ProTaper Ultimate showed better centering ability. The study of root canal wear, transportation, and centering ability of endodontic mechanical files may help the clinician make a more conscious selection of suitable systems towards different challenging anatomies.

## Figures and Tables

**Table 1:** Sequence of instruments selected from the different rotatory systems, apical size, and taper.

	Pathfiles	File/Apical Size/Taper	
ProTaper Next	Proglider—0.16/(* VT)	X1—0.17 (* VT)	X2—0.25 (* VT)
TruNatomy	TN glider—0.17/0.02		TN prime—0.26/0.04
Race Evo	R1—Glidepath—0.15/0.04		R2—0.25/0.04
ProTaper Ultimate	Slider—0.16/0.02	Shaper—0.20/0.04	F1—0.20/0.07

* VT–Variable taper.

**Table 2:** Transversal diameter of the files used.

	Taper	Tip 0	3 mm	5 mm
PTN X2	variable	0.25	0.43	≅0.56
TN Prime	0.04	0.26	0.38	0.46
RE R2	0.04	0.25	0.37	0.45
PTU F1	0.07	0.20	0.41	0.55

**Table 3:** Total mesio-distal wear in millimeters at 3 mm. Values are median (minimum; maximum).

	Straight Canals	Moderate Curvatures	Severe Curvatures
PTN	0.181 (0.132; 0.270)	0.153 (0.045; 0.430)	0.211 (0.119; 0.339)
TN	0.177 (0.045; 0.365)	0.098 (0.024; 0.198)	0.149 (0.047; 0.266)
RE	0.389 (0.326; 0.426)	0.291 (0.127; 0.365)	0.340 (0.262; 0.491)
PTU	0.135 (0.060; 0.380)	0.270 (0.140; 0.360)	0.190 (0.090; 0.630)

**Table 4:** Total mesio-distal wear in millimeters at 5 mm. Values are median (minimum; maximum).

	Straight Canals	Moderate Curvatures	Severe Curvatures
PTN	0.146 (0.041; 0.236)	0.195 (0.101; 0.421)	0.222 (0.414; 0.093)
TN	0.253 (0.041; 0.309)	0.121 (0.034; 0.194)	0.159 (0.025; 0.340)
RE	0.335 (0.106; 0.408)	0.386 (0.194; 0.479)	0.330 (0.145; 0.386)
PTU	0.170 (0.140; 0.240)	0.270 (0.200; 0.370)	0.236 (0.050; 0.390)

**Table 5:** Canal transportation in millimeters at 3 mm. Values are median (minimum; maximum).

	Straight Canals	Moderate Curvatures	Severe Curvatures
PTN	−0.009 (−0.080; 0.128)	0.060 (−0.123; 0.369)	0.056 (−0.251; 0.238)
TN	−0.057 (−0.171; 0.019)	0.012 (−0.184; 0.164)	0.045 (−0.083; 0.280)
RE	0.092 (−0.051; 0.172)	−0.041 (−0.168; 0.128)	0.077 (−0.156; 0.120)
PTU	0.005 (−0.220; 0.100)	−0.175 (−0.500; 0.020)	−0.009 (−0.160; 0.450)

**Table 6:** Canal transportation in millimeters at 5 mm. Values are median (minimum; maximum).

	Straight Canals	Moderate Curvatures	Severe Curvatures
PTN	0.025 (−0.060; 0.104)	−0.021 (−0.283; 0.333)	−0.039 (−0.317; 0.300)
TN	−0.076 (−0.246; 0,014)	−0.022 (−0.159; 0.115)	0.036 (−0.150; 0.145)
RE	−0.114 (−0.206; −0.015)	0.045 (−0.280; 0.182)	0.005 (−0.034; 0.119)
PTU	0.035 (−0.16; 0.09)	−0.115 (−0.26; 0.07)	−0.010 (−0.21; 0.11)

**Table 7:** Centering ability at 3 mm. Values are median (minimum; maximum).

	Straight Canals	Moderate Curvatures	Severe Curvatures
PTN	0.357 (0.245; 0.905)	0.301 (0.041; 0.811)	0.452 (0.012; 0.920)
TN	0.406 (0.207; 0.951)	0.080 (0.013; 0.265)	0.252 (0.007; 0.921)
RE	0.504 (0.143; 0.777)	0.541 (0.117; 0.900)	0.563 (0.371; 0.881)
PTU	0.500 (0.260; 1.500)	0.473 (0.080; 1.330)	0.720 (0.000; 1.500)

**Table 8:** Centering ability at 5 mm. Values are median (minimum; maximum).

	Straight Canals	Moderate Curvatures	Severe Curvatures
PTN	0.269 (0.063; 0.844)	0.091 (0.000; 0.992)	0.268 (0.022; 0.695)
TN	0.367 (0.016; 0.537)	0.229 (0.025; 0.700)	0.364 (0.099; 0.821)
RE	0.468 (0.329; 0.731)	0.634 (0.196; 0.818)	0.774 (0.295; 0.986)
PTU	0.400 (0.313; 1.429)	0.331 (0.071; 1.700)	0.647 (0.087; 1.786)

## Data Availability

The original contributions presented in this study are included in the article. Further inquiries can be directed to the corresponding authors.
